# Robotic Assistance in Unicompartmental Knee Arthroplasty Results in Superior Early Functional Recovery and Is More Likely to Meet Patient Expectations

**DOI:** 10.1155/2021/4770960

**Published:** 2021-07-14

**Authors:** Meredith P. Crizer, Amer Haffar, Andrew Battenberg, Mikayla McGrath, Ryan Sutton, Jess H. Lonner

**Affiliations:** Rothman Orthopaedic Institute at Thomas Jefferson University, Philadelphia, PA, USA

## Abstract

Robotic technology has reduced the errors of implant alignment in unicompartmental knee arthroplasty (UKA), but its impact on functional recovery after UKA is poorly defined. The purpose of this study was to compare early functional recovery, pain levels, and satisfaction in UKA performed with either robotic assistance or conventional methods. A retrospective analysis was performed on 89 matched consecutive patients who underwent outpatient UKA by a single physician using either conventional instruments (*n* = 39) or robotic methods (*n* = 50), with otherwise identical perioperative protocols. Outcomes studied included Lower Extremity Functional Score (LEFS), new Knee Society Score (KSS), Knee Injury and Osteoarthritis Outcome Score for Joint Replacement (KOOS-JR.), VR/SF-12, Visual Analog Scale (VAS) pain scores, and perioperative opioid consumption. Patients in the robotic cohort had superior early functional outcomes, with greater LEFS (conventional = 23; robotic = 31) at 1 week post-op (*p*=0.015) and KOOS-JR (conventional = 74; robotic = 81) at up to 6 months post-op (*p*=0.037); these two values remained statistically significant after mixed-model regression analysis (*p*=0.010; *p*=0.023), respectively. At 1 year post-op, expectations were more likely to be met in those who received robotic assistance (*p*=0.06). No differences were reported with respect to postoperative opioid usage (*p*=0.320), reoperations (*p*=1.00), and complications (*p*=0.628). Robotic-assisted UKA resulted in more rapid recovery and less early postoperative pain and were more likely to meet expectations than conventional UKA, although functional differences equilibrated by 1 year postoperatively. Further follow-up is necessary to determine if implant durability is impacted by robotics.

## 1. Introduction

Unicompartmental knee arthroplasty (UKA) is an effective alternative to total knee arthroplasty (TKA) in patients with osteoarthritis (OA) localized to a single tibiofemoral compartment of the knee [1–3]. UKA has several potential advantages over TKA including faster recovery times, superior postoperative function, more physiological gait, shorter hospital stay, lower blood loss, decreased postoperative morbidity, reduced postoperative opioid consumption, and lower perioperative costs [4–6].

Despite these benefits and the remarkable mid- and long-term success and durability of UKA performed by high-volume UKA surgeons [7–11], UKA failures, and the need for revision related to component malposition, may occur more frequently compared to TKA, particularly in the hands of lower volume surgeons [3, 12–17]. Nationwide insurance statistics and international joint arthroplasty registries have demonstrated that UKA's fail and require revision at a substantially greater rate than TKA at all time points [3, 18, 19]. In a multicenter analysis of 418 failed UKAs, it was reported that 12% of aseptic failures of UKA were attributed to faulty implantation and malpositioning of components and that 49% of these failures occurred within 5 years postoperatively [20]. Other studies have shown that excessive tibial slope and small errors of 2° or 3° in the coronal plane increase the risk of mechanical failure after UKA [14–17]. Conventional methodology for UKA has been shown to result in outliers of greater than 2° in as many as 40–60% of procedures [12, 21].

Robotic assistance was introduced to improve the accuracy of implantation in UKA, and indeed, a number of studies have shown that to be the case [22–28]. Studies have shown reduction in surgical error with robotic assistance compared to manual methods of surface preparation [23, 24, 26]. It has been posited that optimizing component alignment and positioning, as well as quantifying soft tissue balance, would lead to improvement in patient outcomes. Nonetheless, it is unknown whether the improved precision and reduced error with robotics impact clinical function and implant survivorship in UKA [29–33]. The purpose of this study is to compare early recovery, pain levels, short-term functional outcomes, and implant durability in patients undergoing UKA using either conventional manual instrumentation or a robotic sculpting tool.

## 2. Materials and Methods

After obtaining institutional review board approval, a retrospective cohort study (level III) of matched consecutive UKA cases operated on by a single surgeon at two facilities was conducted. UKA was performed with conventional instrumentation (*n* = 39) or robotic technology (*n* = 50) depending on the geographic location of the procedure and access to robotic technology at one center or another. Surgeries were performed consecutively from August 2015 to April 2018. In the conventional cohort, ZUK implants (Smith and Nephew, Memphis, TN) were used while Stride implants (Smith and Nephew, Memphis, TN) were used in the robotic cohort. The primary investigator's criteria for UKA include the presence of medial unicompartmental arthritis, as well as minimum flexion arc of 90°, flexion contracture of less than 5°, varus deformity of less than 10°, a competent anterior cruciate ligament, and absence of pain or exposed bone in the patellofemoral and lateral compartments. Patients were included in the analysis if they underwent outpatient unilateral medial UKA, and those who underwent lateral UKA and simultaneous bilateral UKA were excluded. Demographics and comorbidities were similar between groups (Table 1).

In all cases, a fixed-bearing fully cemented UKA was utilized. In the robotic cohort, the Navio handheld robotic sculptor (Smith and Nephew, Memphis, TN) was used. Navio is an image-free semiautonomous robotic system that utilizes optical-based navigation with an imageless system to provide 3D morphed images. The precision of Navio has been shown to result in mean rotational errors within 1.04°–1.88° and 1.48°–1.98° of the femoral and tibial implants, respectively, with mean femoral and tibial transitional errors reported to be within 0.72–1.29 mm and 0.79–1.27 mm, respectively [24]. It relies on intraoperative surface and limb mapping and registration to plan condylar bone resections and quantify soft tissue balancing. The system provides protective control against inadvertent bone removal by modulating the exposure and speed of the motorized bur. In the conventional instrumentation cohort, proximal tibial resection was performed with an extramedullary tibial cutting guide, and a spacer block technique was used for femoral preparation.

In the robotic system studied, an algorithm is followed to establish the tightness or laxity in the hemicompartment after virtual positioning of the components. After arthrotomy and removal of the medial osteophytes, a valgus stress is applied to the knee (in the case of medial UKA) as it is passively moved from full extension to deep flexion. After surface mapping and 3-dimensional planning of implant sizes, their position and orientation are “virtually” established. A graphic representation of gap spacing through an entire range of motion is created. Adjustments can be made in the virtual positioning of the components—including tibial slope and depth of resection, or femoral component flexion, anteriorization, and distalization—to achieve the desired soft tissue balance across the entire flexion arc. The goal is to adjust the implant positions and orientations such that the gaps in extension and flexion are balanced according to surgeon preferences, with roughly 2 mm of laxity between the components through a full arc of motion, and avoiding overcorrection of alignment into the opposite compartment.

In the conventional technique used in this study, the distal femoral resection was based on the tibial cut, with the goal of leaving 2 mm of laxity between the articulating surfaces of the femoral and tibial trial components, confirmed by a 2 mm spacer device.

Perioperative protocols for anesthesia, surgical incision, periarticular capsular injection, physical therapy, pain management, and overall care were otherwise identical. At our institution, postoperative rehabilitation includes immediate weight bearing as tolerated with a walker, followed by a transition to a cane at the discretion of the physical therapist. Upon restoration of adequate quadriceps strength and balance, patients were allowed to ambulate without assistance. Self-directed exercises began on the day of surgery, and formal physical therapy was initiated within 4 days after surgery, including range of motion and isometrics.

The primary patient-reported outcomes (PROs) studied were Knee Injury and Osteoarthritis Outcome Score for Joint Replacement (KOOS-JR), new Knee Society Score (KSS), Lower Extremity Functional Score (LEFS), Visual Analog Scale (VAS) pain scores (0–100), and Veterans RAND 12-Item Health Survey (VR-12/SF-12) including both mental and physical health scores. The LEFS, new Knee Society Score, and KOOS-JR surveys are reliable, validated, and responsive measurements of lower body and knee health outcomes while VAS and VR-12/SF-12 are accurate and precise measurements of pain and physical and mental health, respectively [34–37]. PROs were collected 30 days preoperatively, and with the exception of new KSS, at 1 week, 6 weeks, 12 weeks, and 2 years postoperatively for VR-12/SF-12 and KOOS-JR. Postoperatively, the new KSS was assessed only at one year postoperatively. VAS pain scores were collected preoperatively and weekly at 1, 2, 3, 6, and 12 weeks. A linear mixed-effects model was used to assess differential change over time between the two groups. Secondary outcomes studied were postoperative opioid consumption up to 3 months after surgery, reoperations, revisions, and postoperative complications.

Mean age, BMI, KOOS-JR, LEFS, new KSS, VR-12/SF-12, and VAS scores were analyzed with *t*-tests for significance. A *p* value of <0.05 was set as the threshold to establish statistical significance of the results. Means of reported VAS pain scores were adjusted for group baseline differences. A linear mixed-effects model was utilized to assess differential changes among patients in the two cohorts.

Total 1 month preoperative and total 3 month postoperative opioid usage were determined using the Pennsylvania Prescription Drug Monitoring Program (PDMP) and New Jersey Prescription Monitoring Program (NJ PMP). These statewide databases have a reported accuracy of up to 97% and collect all information for filled prescriptions of all controlled substances. Opioid usage was reported in morphine milligram equivalents (MME).

## 3. Results

KOOS-JR scores increased for both cohorts over time. The robotic cohort had higher preoperative KOOS-JR scores than the conventional cohort (*p*=0.029), and remained significantly higher at 6 weeks (*p*=0.001) and 6 months (*p*=0.0037) following surgery. While the robotic cohort had higher KOOS-JR scores at 2-year follow-up, the difference between the two cohorts was not statistically significant at that timeframe (*p*=0.469) (Table 2).

Both cohorts experienced an increase in LEFS throughout the duration of the study. LEFS scores were significantly higher in the robotic cohort than in the conventional group at 1 week post-op but were similar at 6 weeks and 12 weeks post-op (Table 2). Mixed-model regressions were run to assess whether the improvement in LEFS and KOOS-JR was attributable to cohort placement. LEFS improved at 6 weeks and 12 weeks post-op (*p* < 0.001 at both time points). Similarly, KOOS-JR increased at 6 weeks, 12 weeks, 6 months, and 1 and 2 years following surgery (*p* < 0.001 at all time points). These regressions showed that the increase in LEFS attributable to robotic assistance is 8.47, 95% CI [2.25; 14.69] (*p*=0.010); for KOOS-JR, these values were 7.26, 95% CI [1.18; 13.33] (*p*=0.023).

Preoperatively, there were no significant differences between groups in baseline total KSS score, or the individual component scores for functional activities, expectations, satisfaction, or symptoms. At 2 years after surgery, there was a significant improvement in all KSS elements compared to preoperative scores, with the patients in the robotic cohort more likely to have had their expectations met (*p*=0.006). Those who received robotic assistance during UKA had higher satisfaction scores than those who did not and this approached statistical significance (*p*=0.068). Otherwise, there were no differences between subsets (Table 3).

There was no significant difference in the mean preoperative ROM in the conventional cohort (113° ± 8°) and in the robotic cohort (116° ± 8°) (*p*=0.054). Likewise, at 2 years after UKA, the ROM in each group was similar (robotic cohort ROM: 131° ± 11°; conventional cohort ROM: 131° ± 8°) (*p*=0.825).

The difference in pre-op VAS pain scores was statistically significant (*p*=0.001) between the two groups, as patients in the robotic-assisted cohort experienced an average VAS score of 44 while patients in the conventional cohort had a VAS pain score of 62. Patients who had robotic UKA experienced less pain at 1 week and 2 weeks post-op, but these findings were not statistically significant (*p*=0.146 and *p*=0.234, respectively). At 3 weeks post-op, patients in the robotic cohort had lower VAS pain scores (26) than those in the conventional cohort (39) (*p*=0.018). These differences equilibrated by 6 weeks post-op (Table 4). No difference was found in 1 month total preoperative narcotic usage nor 3 month total postoperative narcotic usage between the two cohorts (*p*=0.282 and *p*=0.320, respectively) (Table 4). VAS scores are reported on a scale of 0–100 while opioid usage is reported in morphine milligram equivalents (MME).

Mixed-model regression analysis was run to control for baseline differences and elucidate whether cohort placement resulted in greater reduction of VAS pain scores over time. VAS scores decreased up until 6 weeks post-op (*p* < 0.001). No difference was found in changes in VAS scores that were attributable to cohort placement (−0.23, 95% CI: −1.37, 0.91, *p*=0.699).

Patients in both the robotic and conventional cohorts had statistically similar physical (SF-12 PCS) and mental health scores (SF-12 MCS) preoperatively (*p*=0.691 and *p*=0.760, respectively). Despite similar baseline measures, at 6 weeks, the robotic cohort had a higher SF-12 PCS score (43 ± 9) than the conventional cohort (39 ± 8), approaching, but not achieving, statistical significance (*p*=0.099) (Table 5).

SF-12 MCS scores were found to be significantly higher in the robotic cohort (55 ± 7) than in the conventional cohort (51 ± 7), (*p*=0.017). Differences in SF-12 PCS equilibrated by 12 weeks. Both groups demonstrated statistically comparable improvement in SF-12 MCS at 6 and 12 weeks.

No patients in our cohort experienced a mechanical failure, infection, or revision after surgery. One patient in the robotic-assisted cohort had a reoperation: irrigation and debridement for traumatic wound dehiscence after a fall two weeks following surgery, while no patients in the conventional cohort had any reoperations. Overall, there was no statistical significance with respect to complications and reoperations between the two cohorts (*p*=0.628 and *p*=1.00, respectively).

## 4. Discussion

Experience with robotic technology for UKA has consistently shown improvements in radiographic alignment [27]. Less certain, and less studied, is the impact of robotic precision on clinical outcomes, postoperative pain, patient satisfaction, and implant durability. This study was an attempt to add additional insight regarding whether UKA performed with robotic technology that quantifies both bone resection parameters and soft tissue balance impacts early outcomes and short-term durability any differently than a matched group of patients undergoing UKA with conventional instrumentation.

Our data show that, in our matched cohorts, robotic assistance during UKA does indeed provide patients with greater early functional recovery up to 6 months following surgery, according to the LEFS and KOOS-JR scores. The LEFS, in particular, has been shown to be extremely responsive to subtle differences early in the postoperative period. Any functional differences in our study equilibrated by 1 year. We found no difference in cumulative 3-month postoperative opioid usage. Finally, we found that patients in the robotic cohort were statistically significantly more likely to have their expectations met at 2-year follow-up. Satisfaction was also higher in the robotic cohort, and this approached statistical significance (*p*=0.068).

A prospective study by Blyth et al. of 139 patients undergoing medial UKA randomized to using either manual conventional cutting instruments or haptic robotic assistance found significant reductions in pain in the initial two months after robotic UKA [29]. The authors found that, from the first postoperative day, up until week 8 after UKA, the median pain scores for the robotic group were 55.4% lower than those observed in the manual surgery group (*p*=0.040). Similarly, VAS pain scores for both of our cohorts decreased significantly up until 6 weeks post-op. However, unlike the Blyth study, while VAS pain scores in our series were lower for patients in the robotic cohort at three weeks post-op, mixed-model regression analysis showed no difference between these two cohorts with respect to decrease in VAS pain scores at measured time intervals. Furthermore, Blyth and colleagues reported that, at three months postoperatively, the robotic group had significantly better Knee Society Scores (KSS) than the conventional group (*p*=0.04), although the KSS may not be an optimal measure of early functional outcomes, and the KSS differences were not apparent at one year after UKA. This is similar to what we found with regard to LEFS and KOOS-JR early in the postoperative period, and similarly, we found no differences in 1-year KSS or KOOS-JR scores. In comparison with those who underwent robotic UKA in the Blyth study, patients who underwent UKA with robotic assistance in our study seemed to have higher KOOS-JR scores. At 1 year post-op, the average KOOS-JR in the robotic-assisted cohort was 84. Using the crosswalk of KOOS-JR to Oxford Knee Scores (OKS) [38], this corresponds to an OKS of 44. In contrast, less than half of the robotic patients in the Blyth study failed to achieve an OKS of 43. Although there was no overall statistical difference between groups, the proportion of patients in the Blyth study achieving a Forgotten Joint Score of >80%—a measure of the patient's awareness of their joint—was almost double in those who underwent UKA with robotic assistance compared to conventional methods (15% versus 8%, *p*=0.265).

At one year postoperatively, a greater proportion of patients receiving robotic-assisted surgery improved their UCLA activity scores (69% versus 52%; *p*=0.06). Importantly, the authors noted that on subgroup analysis of 35 patients considered to be highly active preoperatively, there was statistically better improvement in function with robotic assistance than with conventional techniques for KSS, Oxford Knee Score, and Forgotten Joint Score (*p*=0.0346) one year after UKA [29], and these superior results persisted at 2-year clinical follow-up [31]. Our study was not adequately powered to review a subset of highly active patients to determine whether we had comparable benefits of robotics in such a cohort.

Other studies have also shown more rapid clinical recovery after UKA performed with robotic assistance compared to conventional methods. In a retrospective review of 28 lateral UKAs, 11 performed with robotic assistance with the same handheld image-free device used in the present study and 17 performed with conventional instruments, Canetti et al. reported a more rapid return to sports with robotic assistance (mean and standard deviation of 4.2 ± 1.8 months compared with 10.5 ± 6.7 months; *p* < 0.01) [33]. However, as the authors point out, surgical approaches varied between the two groups, recall bias may have existed when retrospectively determining interval to return to sport, and it is unclear whether perioperative protocols differed between the groups in that study [33]. Kayani et al. reported on a prospective consecutive cohort study of 146 patients that underwent medial UKA using either conventional jig-based instrumentation or robotic arm assistance. Robotic arm-assisted UKA was associated with reduced postoperative pain (*p* < 0.001), decreased opiate analgesia requirements (*p* < 0.001), shorter time to straight leg raise (*p* < 0.001), decreased number of physiotherapy sessions (*p* < 0.001), and increased maximum knee flexion at discharge (*p* < 0.001) [39]. While these outcomes are encouraging, it is unclear whether pain levels were similar prior to surgery and thus whether the significant differences in postoperative pain levels were truly clinically significant or an equivalent change between groups compared to preoperative pain levels. Furthermore, it is unclear whether perioperative pain management protocols were similar between groups. If they were similar, the reduced opiate requirements in their study is worth highlighting, particularly given that both our study and the study by Blyth et al. found no differences [29].

We found no difference between groups in cumulative postoperative opioid prescriptions in the three months following surgery. This finding is in agreement with the findings of Blyth et al. [29]. However, one weakness in our methodology is that we utilized a statewide database, the Pennsylvania Prescription Drug Monitoring Program (PDMP), to record opioid usage. While this database has a reported accuracy of up to 97% with respect to quantity prescribed, we were unable to track or study patient opioid usage. Moreover, with narcotics being prescribed to patients in accordance with standard protocols, the quantities that were obtained from the statewide database may not accurately reflect postoperative narcotic use, particularly for those who abstain or are low-level users. Further studies which investigate opioid usage between these two methods of UKA are needed.

At this time, mid- and long-term survivorship studies are lacking in robotic-assisted UKA. Nonetheless, several series comparing revision rates and durability to historical controls, registry and insurance databases, and comparable cohorts have shown encouraging results at short term to midterm [27, 30, 31, 40]. A prospective randomized trial found that at 2-year follow-up while no revisions were necessary in the robotic-assisted group, there were 2 revisions (2.8%) in the manual group and more radiolucencies beneath the components in the conventional group [31]. In a matched case-control study of 160 UKA performed with either Navio or a conventional technique using the identical implant (HLS Uni Evolution, Tornier®), Batailler et al. found that, at a mean follow-up of 19.7 months for the robotic-assisted group and 24.2 months for the control group, 5% (*n* = 4/80) of patients in the robotic-assisted UKA group and 9% (*n* = 7/80) in the conventional UKA group required revision to TKA (n. s. d.) [27]. In the conventional group, 86% of revisions were due to component malposition or limb malalignment, compared to none in the robotic-assisted group [27]. In a clinical study of 128 patients undergoing UKA with NAVIO robotic assistance, Battenberg et al. reported that the overall survivorship of the knee implant among novice users was 99.2% (95% confidence interval: 94.6 to 99.9%) after a mean of follow-up period of 2.3 years, compared to a reference survival rate of 95.7% from the Australian registry and 92.6% from a US Medicare database [3, 18, 30].

Similarly, our matched study of UKA performed with conventional instruments or robotics had a low revision rate in both cohorts, with no difference in failure rates, albeit further follow-up will be necessary to clearly determine if there are longer-term benefits to the improved accuracy of implantation and quantified soft tissue balance with robot assistance.

It is unclear why some of the outcome parameters studied in our series were different between the robotic and conventional groups up until 1-year follow-up, given generally otherwise identical surgical approaches and perioperative protocols. One possible explanation may be that quantifying soft tissue balance may impact early function. Robotic assistance during UKA utilizes optical motion capture technology to provide real-time medial and lateral gap balance measurements under valgus/varus strain to induce ligament tension through the arc of flexion [22]. These parameters allow surgeons to achieve the desired ligamentous tension and limb alignment by fine-tuning implant positioning [22].

As with all retrospective studies, ours has several potential limitations. First, ours was a matched cohort of consecutive cases performed with or without robotic assistance based on geographic location and robotic access according to hospital selection. While a randomized prospective study may have been better to eliminate the potential for selection bias, we feel that we were able to mitigate some of the deficiencies of a retrospective cohort study since demographics, patient socioeconomics, preoperative functional scores, and range of motion were matched, we used fixed-bearing cemented UKA implants of similar design in each group, and perioperative protocols such as physical therapy and pain management were identical. A second potential weakness which could serve as a confounding variable for this study is that the operative surgeon has extensive experience with both conventional and robotic techniques, which may have reduced the differences between the two groups. It is possible that surgery performed by less experienced surgeons, or a multicentered study, may or may not yield different results that favor one method over another, in terms of both clinical outcomes and implant survivorship. For instance, Karia et al. have shown that robotic assistance neutralizes surgeon inexperience and improves alignment measures compared to persistent errors with conventional methods in the hands of inexperienced surgeons [41]. We would encourage a study similar to ours by surgeons with variable operative experience with either conventional or robotic techniques to determine the impact of advanced precision on functional outcomes in UKA in their hands. Third, due to matching of the cohorts, our sample size was limited to 39 in the conventional instrumentation group and 50 in the robotic technology arm, which may have increased the risk of type II error. Fourth, our study did not look at the impact of component alignment on functional outcomes, as we had a relatively high incidence of postoperative short radiographs in both groups which were slightly rotated, thus rendering radiographic measurements of alignment inaccurate. Nonetheless, others have studied implant alignment comparisons between conventional and various robotic systems. Fifth, our follow-up was only one year after surgery. While our results show that patients in both groups experienced similar functional outcomes and implant durability at 1-year follow-up, further follow-up is necessary to elucidate whether either technique suffers increased failures at mid- and longer-term follow-up.

## 5. Conclusion

Robotic-assisted UKA had superior functional outcomes up until 6 months following surgery, with differences equilibrating between the two cohorts by 1 year post-op. While the robotic cohort had lower VAS pain scores at 3 weeks post-op, mixed-model regression analysis showed this decrease was not attributable to cohort placement. We also noticed no difference in cumulative postoperative opioid prescriptions, although we were unable to determine precise opioid usage. Those who received robotic assistance were more likely to have their expectations met and satisfaction was tending to be higher in the robotic cohort as well. Despite these promising early results, further mid- and long-term studies are needed to better assess whether robotic-assisted UKA provides longer-term benefits on clinical functionality, implant durability, and patient satisfaction. Otherwise, if these outcome metrics are not appreciably impacted by using robotic technology, its broader use will only be considered if robotics can be shown to be cost-effective and time-efficient and eliminate instrument tray burden.

## Figures and Tables

**Table 1:** Summary of cohort demographics.

Dependent variable	Conventional	Robotic	*p* value
	*N* = 39	*N* = 50	
Gender			1.000
** **Female	17 (43.6%)	21 (42.0%)	
** **Male	22 (56.4%)	29 (58.0%)	
Age (years)	58 (13)	63 (11)	0.073
BMI	28.3 (4.06)	28.1 (4.45)	0.783
CCI	0.37 (0.63)	0.52 (0.93)	0.367
CCI (age adjusted)	1.76 (1.58)	2.34 (1.64)	0.099

Values are reported in means and standard deviations, with the exception of gender, which is reported in means and percentages relative to the entire cohort. CCI: Charlson Comorbidity Index.

**Table 2:** A summary of functional outcomes.

Dependent variable	Conventional	Robotic	*p* value
LEFS 1 week	23 (11)	31 (14)	0.015
LEFS 6 weeks	55 (10)	59 (13)	0.181
LEFS 12 weeks	59 (12)	62 (12)	0.324
Pre-op KOOS-JR^2^	49 (12)	57 (14)	0.029
6-week KOOS-JR	67 (8)	76 (9)	0.001
12-week KOOS-JR	68 (10)	74 (13)	0.131
6-month KOOS-JR	74 (10)	81 (11)	0.037
1-year KOOS-JR	77 (13)	84 (15)	0.100
2-year KOOS-JR	82.9 (20)	86.2 (17)	0.469

Values are reported in means and standard deviations. LEFS: Lower Extremity Functional Scale; KOOS-JR: Knee Injury and Osteoarthritis Outcome Score for Joint Replacement.

**Table 3:** A summary of preoperative, 2 year postoperative new Knee Society Scores, and 2 year postoperative VAS satisfaction scores.

Dependent variable	Conventional	Robotic	*p* value
Pre-op KSS functional activities (0–100)	47 (18)	48 (18)	0.811
Pre-op KSS patient expectations (0–15)	14 (3)	13 (3)	0.390
Pre-op KSS satisfaction (0–40)	16 (6)	16 (7.5)	0.552
Pre-op KSS symptoms (0–25)	9 (6)	12 (6)	0.148
2-year post-op KSS functional activities (0–100)	74 (19)	82 (17)	0.208
2-year post-op KSS patient expectations (0–15)	8 (2)	11 (3)	0.006
2-year post-op KSS satisfaction (0–40)	28 (10)	34 (9)	0.068
2-year post-op KSS symptoms (0–25)	20 (5)	21 (5)	0.269
2-year post-op VAS satisfaction	86 (20)	92 (18)	0.301

**Table 4:** A summary of VAS scores and opioid usage.

Dependent variable	Conventional	Robotic	*p* value
Pre-op VAS pain	62 (18)	44 (22)	0.001
1-week post-op VAS pain	60 (18)	53 (18)	0.146
2-week post-op VAS pain	46 (20)	39 (15)	0.234
3-week post-op VAS pain	39 (19)	26 (16)	0.018
6-week post-op VAS pain	23 (16)	18 (16)	0.370
12-week post-op VAS pain	21 (21)	16 (16)	0.552
Pre-op opioid usage (MME)	6 (7)	8(8)	0.282
Post-op opioid usage (MME)	660 (237)	714 (268)	0.320

**Table 5:** Preoperative and postoperative SF-12 PCS and SF-12 MCS values.

Dependent variable	Conventional	Robotic	*p* value
Pre-op SF-12 PCS	36 (8)	35 (9)	0.691
6-week SF-12 PCS	39 (8)	43 (9)	0.099
12-week SF-12 PCS	45 (8)	48 (7)	0.140
2-year SF-12 PCS	48 (10)	50 (11)	0.601
Pre-op SF-12 MCS	57 (8)	56 (11)	0.760
6-week SF-12 MCS	56 (8)	57 (7)	0.658
12-week SF-12 MCS	58 (5)	57 (9)	0.644
2-year SF-12 MCS	51 (7)	55 (7)	0.017

Values are reported in means and standard deviations.

## Data Availability

The data used to support the findings of this study have not been made available.
